# Multi-slice computed tomography assessment of bronchial compression with absent pulmonary valve

**DOI:** 10.1007/s00247-014-2898-z

**Published:** 2014-04-05

**Authors:** Yu-Min Zhong, Richard B. Jaffe, Jin-Fen Liu, Ai-Min Sun, Wei Gao, Qian Wang, Ming Zhu, Hai-Sheng Qiu, Walter E. Berdon

**Affiliations:** 1 Department of Radiology, Shanghai Children’s Medical Center and Shanghai Jiao Tong University Medical School, Shanghai, China; 2 Department of Cardiology, Shanghai Children’s Medical Center and Shanghai Jiao Tong University Medical School, Shanghai, China; 3 Department of Cardiothoracic Surgery, Shanghai Children’s Medical Center, No.1678 Dong Fang Road, Shanghai, 200127 China; 4 Department of Medical Imaging, Primary Children’s Medical Center, Salt Lake City, UT USA; 5 Department of Radiology, Children’s Hospital of New York, New York, NY USA

**Keywords:** Absent pulmonary valve, Tracheobronchial compression, Multi-slice computed tomography, Bronchoscopy, Children, Congenital heart disease

## Abstract

**Background:**

Absent pulmonary valve is a rare cardiovascular anomaly that can result in profound tracheobronchial compression.

**Objective:**

To demonstrate the advantage of multi-slice CT in diagnosing tracheobronchial compression, its severity as related to the adjacent dilated pulmonary arteries, and associated lung and cardiac lesions.

**Materials and methods:**

We included children with absent pulmonary valve who were reviewed by multi-slice CT during a 17-year period. The number and locations of stenoses and lung lesions were noted and the severity of stenosis was categorized. The diameter of the pulmonary artery was measured and associated cardiac defects were demonstrated.

**Results:**

Thirty-one children (14 girls and 17 boys) were included. Of these, 29 had ventricular septal defect and 2 had an intact ventricular septum. Twenty-nine children (94%) had tracheobronchial compression, judged to be mild in nine children (31%), moderate in 10 (34%) and severe in 10 (34%). The different locations of the stenosis (carina, main bronchi, lobar and segmental bronchi) were observed. And the number and location of lung lesions demonstrated that the right middle and left upper and lower lobes were often affected. The diameter of the pulmonary artery in these children was well above normal published values, and Spearman rank correlation analysis showed a correlation between the size of the pulmonary artery and the severity of the tracheobronchial stenosis. Nineteen children (61%) underwent surgery and 4 of these children had a multi-slice CT post-operative follow-up study.

**Conclusion:**

Absent pulmonary valve can cause significant morbidity and mortality in children. Multi-slice CT can accurately depict areas of tracheobronchial compression, associated lung lesions and cardiac defects, helping to direct the surgeon.

## Introduction

Absent pulmonary valve is a rare cardiovascular anomaly that occurs with ventricular septal defect (absent pulmonary valve with tetralogy of Fallot or with double-outlet right ventricle) or much less commonly with an intact septum (isolated absent pulmonary valve). Characteristic features of absent pulmonary valve with ventricular septal defect include varying degrees of right ventricular outflow tract obstruction, profoundly dilated branch pulmonary arteries, absent ductus arteriosus and association with DiGeorge syndrome and 22q11.2 deletion syndrome. Absent pulmonary valve with ventricular septal defect is often defined as absent pulmonary valve syndrome or tetralogy of Fallot variant. Absent pulmonary valve with an intact ventricular septum can present with patent ductus arteriosus, right ventricle enlargement, infundibular prominence and dilatation of the main pulmonary artery but not the branch pulmonary arteries [1]. Absent pulmonary valve, especially absent pulmonary valve syndrome, can lead to profound bronchial and carina compression caused by the adjacent dilated pulmonary arteries. Usually patients with tracheobronchial compression have respiratory illness [2]. The purpose of this study is to demonstrate the advantage of multi-slice CT in diagnosing tracheobronchial compression, its severity as related to the adjacent pulmonary arteries, and associated lung and cardiac lesions, all helpful information for management decisions.

## Materials and methods

This retrospective research study was compliant with the Health Insurance Portability and Accountability Act and was approved by our tertiary care institutional review board. From Jan. 1, 2006, to Jan. 31, 2013, we identified 40 children with absent pulmonary valve (17 boys, 23 girls). Patient ages ranged from 1 month to 7 years; the median age was 6 months. Thirty-one of these children (14 girls and 17 boys) underwent multi-slice CT and form the basis of this report (Table 1). Multi-slice CT findings were recorded separately by two radiologists and discrepancies were resolved by independent review. We noted the number and location of stenoses (carina, main bronchi, lobar bronchi, segmental bronchi). The severity of stenosis was categorized as mild (less than 25%), moderate (25–75%) and severe (more than 75%) compared with the diameter of the adjacent normal airway using lung window (window width 1,500 HU, window level −500 HU) [3]. We identified the number and location of lung lesions (atelectasis, emphysema). The diameters of the right and left pulmonary arteries were measured in the cranio–caudal plane and normalized to body surface area.

**Table 1:** Clinical information in 31 children with absent pulmonary valve

Clinical information	APV with VSD	APV with intact ventricular septum
Cases	29	2
Heart murmur as a main complaint	29	1
Associated with respiratory illness	17	1
Tracheobronchial compression	28	1
Operation (with pulmonary angioplasty)	18	1
Death (after operation)	2	0
Follow-up multi-slice CT	4	0

*APV* absent pulmonary valve, *VSD* ventricular septal defect

A 16-slice CT system (Light Speed, GE Healthcare, Milwaukee, WI) was used in all 31 cases. Imaging parameters were as follows: 120 kV, 100–200 mA, 0.625 collimation, 5.62 mm/s table speed, rotation speed 0.5 and 0.3-mm reconstruction interval. No cardiac gating was performed using nonionic contrast agent (2 ml/kg of iopamidol, 400 mg/ml; Bracco, Milan, Italy) or iohexol (GE Healthcare, London, UK) injected through a peripheral vein. Whenever an arm vein was used, CT was performed in the caudo–cranial direction to decrease contrast-medium-related artifacts and to achieve homogeneous contrast enhancement. Contrast agent was power-injected at a rate of 0.8–2.5 ml/s according to the scan range. Scan delay was 13–18 s depending on the site of injection. The CT raw data were processed on an off-line workstation (Advantage Windows 4.2; GE Healthcare, Milwaukee, WI) with maximum-intensity projection or minimum-intensity projection algorithms or with volume-rendering technique. A post-operative multi-slice CT study was performed in 4 children. Bronchoscopy was performed in 7 of the 31 children and was correlated with CT findings.

Statistical analysis was performed using SPSS version 13.0. Spearman rank correlation analysis was used to demonstrate the correlation between the size of the pulmonary artery and the severity of tracheobronchial stenosis.

## Results

Of the 31 children with absent pulmonary valve who underwent multi-slice CT, 29 had a ventricular septal defect (27 with tetralogy of Fallot and 2 with double-outlet right ventricle); two of the 31 children had an intact ventricular septum (one with tricuspid atresia and one with atrial septal defect). Multi-slice CT also clearly demonstrated the associated cardiovascular defects (atrial septal defect and patent ductus arteriosus, etc.).

**Fig. 1:**
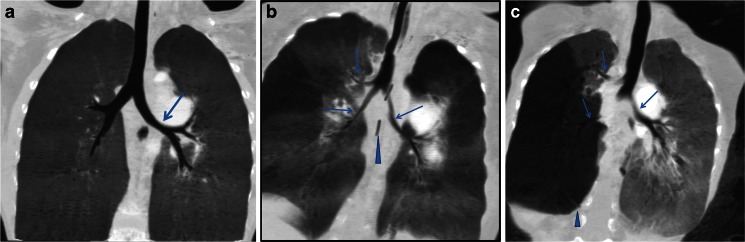
Multi-slice CT findings of severity of tracheobronchial compression. **a** Absent pulmonary valve with tetralogy of Fallot in a 7-year-old boy. The multi-slice CT coronal minimum-intensity projection demonstrates mild left bronchial compression (*arrow*). **b** Absent pulmonary valve syndrome with tetralogy of Fallot in a 4-month-old girl. Multi-slice CT coronal minimum-intensity projection reconstruction demonstrates moderate right and left bronchial compression (*arrows*). The two vertical black lines are part of an esophageal tube (*arrowhead*). **c** Absent pulmonary valve syndrome with tetralogy of Fallot in a 10-month-old girl. Multi-slice CT coronal minimum-intensity projection reconstruction demonstrates severe right tracheal bronchus stenosis and right bronchial compression and moderate left bronchus stenosis (*arrows*). Right middle lobar and lower lobar emphysema are present (*arrowhead*)

**Fig. 2:**
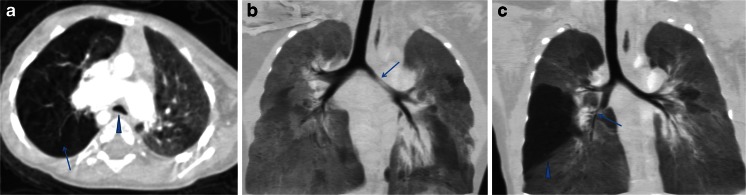
Multi-slice CT findings of location of tracheobronchial compression. **a** Absent pulmonary valve syndrome with tetralogy of Fallot in a 10-month-old girl. Axial multi-slice CT image demonstrates carina and right main bronchus compression; the carina is shaped like an inverted “V” (*arrowhead*). Right upper lobar emphysema is present (*arrow*). **b** Absent pulmonary valve syndrome with tetralogy of Fallot in a 13-month-old boy. Multi-slice CT coronal minimum-intensity projection reconstruction demonstrates moderate left bronchial stenosis (*arrow*). **c** Absent pulmonary valve syndrome with tetralogy of Fallot in a 3-month-old boy. Multi-slice CT coronal minimum-intensity projection reconstruction demonstrates right intermediate bronchial stenosis (*arrow*) and right middle lobe obstructive emphysema (*arrowhead*)

Twenty-nine children had tracheobronchial stenosis (94%). Of these 29 children, 31% (9/29) had mild stenosis, 34% (10/29) had moderate stenosis and 34% (10/29) of cases had severe bronchial stenosis (Fig. 1) (Table 2). The different locations of stenosis (carina, main bronchi, lobar and segmental bronchi) were observed. Forty-five percent (14/31) of the children had carina compression, 77% (24/31) had main bronchi compression and 65% (20/31) had lobar or segmental bronchial compression (Fig. 2). The number and location of lung lesions (atelectasis, emphysema) was also identified. In 29 children who had tracheobronchial stenosis, the right middle lobe, left upper lobe and left lower lobe were most often affected (Table 2).

**Table 2:** Multi-slice CT findings regarding severity of bronchial stenosis in 31 children with absent pulmonary valve (APV)

Clinical information	APV with ventricular defect	APV with intact ventricular septum
Diagnosed by multi-slice CT	29	2
Obvious dilatation of MPA	0	2
Dilatation of RPA and/or LPA	29	1
Dysplasia of LPA	2	0
Number and location of stenoses		
Carina	14	0
Main bronchi	23	1
Lobar and segmental bronchi	20	0
Number and location of lung lesions		
Right upper lobe	9	0
Right middle lobe	22	2
Right lower lobe	7	0
Left upper lobe	22	1
Left lower lobe	14	1
Severity of bronchial stenosis		
Mild (<25%)	9	0
Moderate (25–75%)	9	1
Severe (greater than 75%)	10	0

*LPA* left pulmonary artery, *MPA* main pulmonary artery, *RPA* right pulmonary artery

**Fig. 3:**
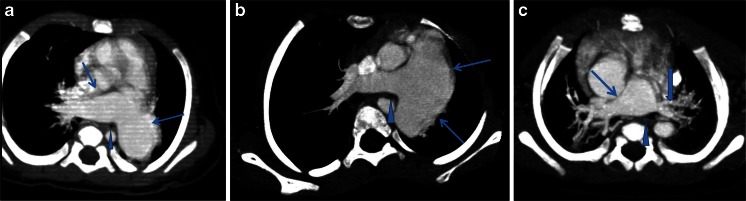
Multi-slice CT findings of absent pulmonary valve with or without ventricular septal defect and compressed bronchus. **a** Tetralogy of Fallot in a 4-month-old girl. Multi-slice CT axial-oblique maximum-intensity projection reconstruction demonstrates right and left pulmonary artery dilatation (*arrows*). Right and left main bronchi are inferior and posterior to the dilated right pulmonary artery. The left bronchus looks displaced posteriorly and compressed (*arrowhead*). **b** Absent pulmonary valve, intact ventricular septum and atrial septal defect in a 17-month-old girl. Multi-slice CT axial-oblique maximum-intensity projection reconstruction demonstrates main pulmonary artery and left pulmonary artery dilatation (*arrows*), which is markedly compressing the left bronchus (*arrowhead*). **c** Absent pulmonary valve syndrome with double-outlet right ventricle, ventricular septal defect, atrial septal defect and left superior vena cava in a 3-month-old boy. Multi-slice CT axial maximum-intensity projection reconstruction demonstrates a dilated right pulmonary artery (*thin arrow*) and hypoplastic left pulmonary artery (*thick arrow*). The proximal left bronchus looks compressed by the dilated right pulmonary artery (*arrowhead*)

In 29/31 children the diameter of the pulmonary artery branches was measured and normalized to body surface area (Table 3). The diameters of right and left pulmonary artery were well above normal published values (normal right pulmonary artery diameter 14 ± 8 mm/m2, left pulmonary artery 12 ± 5 mm/m2) [4]. In 2/31 children with ventricular septal defect, left pulmonary artery hypoplasia was present. In the two children with intact ventricular septum, the main pulmonary artery was dilated (Fig. 3). The results from Spearman rank correlation analysis showed a correlation between the size of the right pulmonary artery and the severity of tracheobronchial stenosis, and multi-slice CT also demonstrated that the right and left main bronchi in most cases were just inferior and posterior to the dilated right pulmonary artery.

**Table 3:** Correlation between the size of the pulmonary branches and the severity of tracheobronchial stenosis

Severity of tracheobronchial stenosis	LPA/BSA (mean ± SD mm/m2)	RPA/BSA (mean ± SD mm/m2)
Absent (2 cases)	32.13 ± 7.58	35.68 ± 8.14
Mild (9 cases)	34.31 ± 14.17	33.61 ± 10.46
Moderate (10 cases)	36.47 ± 17.82	41.94 ± 15.79
Severe (10 cases)	44.06 ± 21.32	50.51 ± 19.85

Spearman rank correlation analysis showed that there is no significant correlation between LPA/BSA and severity of tracheobronchial stenosis. There is a significant correlation between RPA/BSA and severity of tracheobronchial stenosis. The correlation coefficient was 0.371. *BSA* body surface area, *LPA* left pulmonary artery, *RPA* right pulmonary artery, *SD* standard deviation

In these 31 children, 58% (18/31) had respiratory illness that included coughing, pneumonia, shortness of breath and recurrent respiratory infection. One child had dyspnea and ventilator support before surgery.

**Fig. 4:**
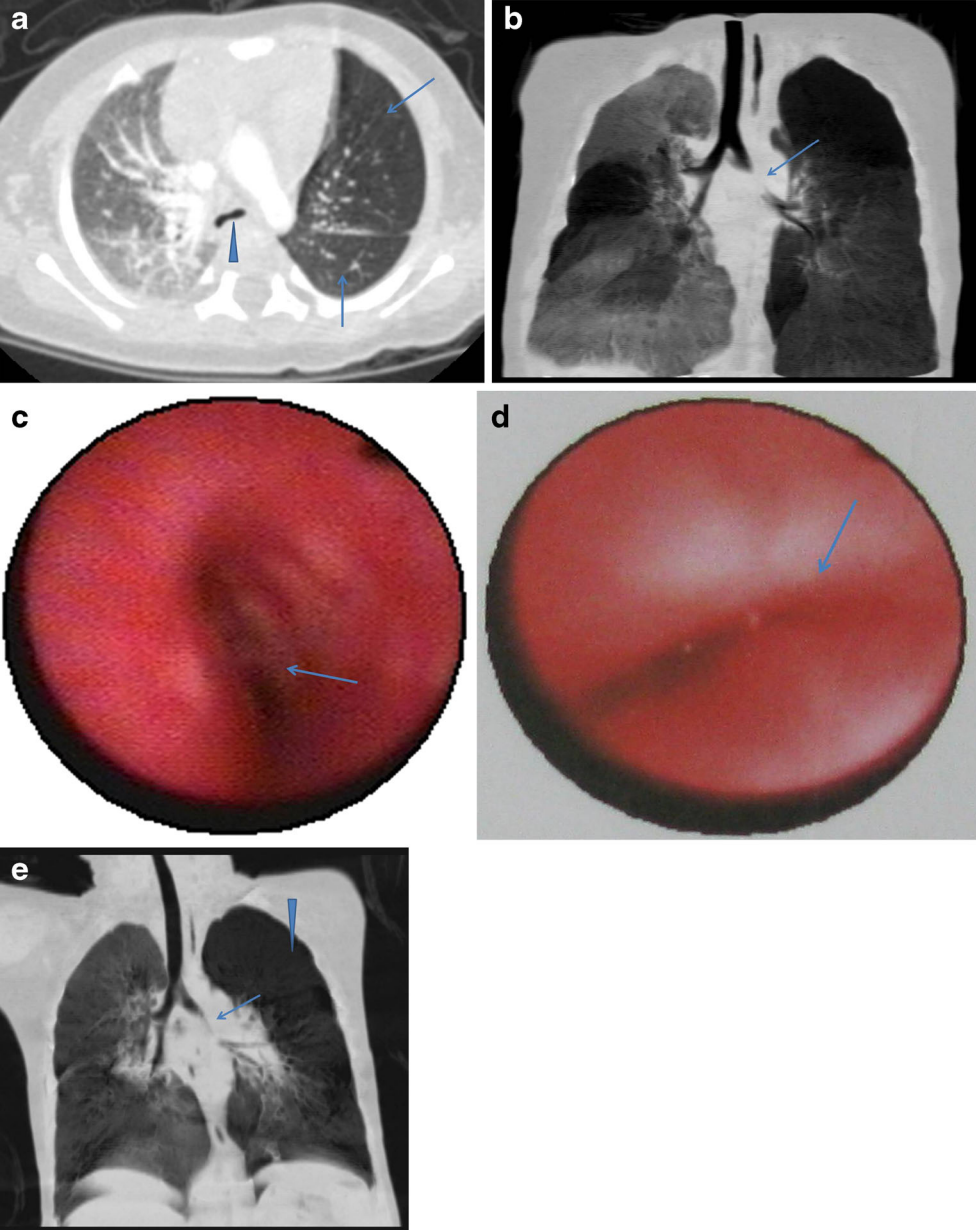
Absent pulmonary valve syndrome with tetralogy of Fallot in a 4-month-old boy. **a** Multi-slice CT axial and (**b**) coronal minimum-intensity projection CT images demonstrate carina (*arrowhead* in **a**) and left main bronchial compression (*arrow* in **b**). Note the obstructive emphysema in the left lung and right middle lobe (*arrows* in **a**). **c, d** Bronchoscopy demonstrates carina compression (*arrow* in **c**) and left main bronchus bronchomalacia (*arrow* in **d**). **e** Multi-slice CT coronal minimum-intensity projection reconstruction 3 months after surgery in the same child, take picture from different angle demonstrates an improved appearance of the left bronchus (*arrow*) and considerable improvement in the right middle lobe and left lung obstructive emphysema (*arrowhead*)

Bronchoscopy was performed in seven children. All seven had moderate to severe bronchomalacia adjacent to dilated pulmonary arteries (Fig. 4) (Table 4).

**Table 4:** Comparison between cross-sectional images and bronchoscopy in 7 children

	CT	Bronchoscopy
Carina compression	5 (posteroanterior stenosis)	3 (malacia)
Right bronchus compression	7	6 (malacia)
Left bronchus compression	7	7 (malacia)

Nineteen children (61%) underwent surgery, which confirmed the diagnosis of absent pulmonary valve. Pulmonary angioplasty was performed in all children (18 underwent pulmonary angioplasty, 1 underwent translocation and pulmonary angioplasty). Two children died of dyspnea and severe pneumonia after surgery.

Four children have been followed with multi-slice CT since their initial surgery. Two of these children had mild stenosis, one had moderate stenosis and one had severe stenosis on CT follow-up study. Partial improved appearance of either the right or left main bronchus was demonstrated in one mild and one severe case (Fig. 4).

## Discussion

Absent pulmonary valve is a rare cardiovascular anomaly [1, 2, 5]. In our series 94% of the children (29/31) had absent pulmonary valve syndrome (93% tetralogy of Fallot variant, 7% double-outlet right ventricle) and 6% (2/31) had absent pulmonary valve with intact ventricular septum (one with tricuspid atresia, one with atrial septal defect) [6–9].

Absent pulmonary valve is associated with significant bronchial and carina compression caused by adjacent pulmonary artery dilatation. In our groups, 94% (29/31) of the children had a significantly dilated branch of the pulmonary arteries, which can result in carina or bronchial compression, and this compression was confirmed by multi-slice CT. Absence or hypoplasia of one pulmonary artery can also occur [1]. Our data presented two cases with hypoplasia of the left pulmonary artery [1, 2], but even these children had narrowing of the left bronchus from adjacent main or right pulmonary artery dilatation.

Children with absent pulmonary valve and an intact ventricular septum usually have a dilatation of the main pulmonary artery rather than the pulmonary branches. If the main pulmonary artery is significantly dilated, compression of the carina and bronchi can occur [1, 2, 5]. In our two patients with absent pulmonary valve and intact septum, the main pulmonary artery was dilated significantly, and in one of these children there was also dilatation of the left pulmonary artery.

MRI can demonstrate tracheobronchial compression but it is time-consuming, and prolonged sedation might not be suitable for infants with severe respiratory illness unless performed under general anesthesia [10, 11].

Although CT remains an ionizing procedure and vascular studies require intravenous contrast injection, the quick examination time and light sedation are beneficial in children with an absent pulmonary valve who have severe respiratory issues [12–14]. Low-dose multi-slice CT with 3-D reconstruction represents an efficient alternative. Although a standard protocol was used in our patients, a non-cardiac-gating protocol can decrease radiation dose. In the axial plane and 3-D reconstruction of multi-slice CT, the locations and severity of tracheobronchial compression and associated lung lesions (atelectasis, emphysema) can be clearly evaluated.

Statistical analysis showed a correlation between the severity of tracheobronchial stenosis and dilatation of right pulmonary artery. And the number and location of lung lesions demonstrated that the right middle lobe, left upper lobe and left lower lobe were most often affected. This was consistent with the findings we observed. In the axial plane, we found that the carina and the right and left main bronchi in most cases were just inferior and posterior to the dilated right pulmonary artery and usually both the right and left bronchi were compressed to an equal degree by the dilated right pulmonary artery. Bilateral segmental atelectasis and obstructive emphysema were clearly visualized [10, 11]. Our data demonstrated that most cases (65%) had moderate to severe bronchial stenosis.

Multi-slice CT, especially low-dose multi-slice CT with 3-D reconstruction, is also a useful modality for evaluation of the tracheobronchial tree after surgery [15]. Four children in our study had post-operative multi-slice CT immediately after surgery. In two children there was an improved appearance of the compressed bronchus.

The sites of extrinsic compression can also be identified by bronchoscopy and tracheobronchography. However these procedures have increased risk in patients with severe respiratory illness in comparison to noninvasive multi-slice CT and MRI. In our group, bronchoscopy performed in seven children prior to surgery confirmed that the tracheobronchial compression seen on CT was from tracheobronchomalacia adjacent to dilated pulmonary arteries.

Children with absent pulmonary valve with ventricular septal defect have more severe clinical symptoms than those with isolated absent pulmonary valve [1]. Eighteen children (58%) in our series had respiratory symptoms including coughing, pneumonia, shortness of breath and recurrent respiratory infection. One child had dyspnea and ventilator support before surgery. Two children died of dyspnea and severe pneumonia after surgery. The evaluation of airway anatomy and possible tracheobronchial stenosis should be performed in pre-operative patients, especially those dependent on ventilators [15]. Multi-slice CT provides helpful information for the surgeon to determine whether reduction pulmonary arterioplasty or translocation is necessary for relief of adjacent tracheobronchial compression. Bronchomalacia can occur from prolonged bronchial compression and tracheobronchial cartilage immaturity before the first 6–9 months of age. Aggressive management as early as possible is needed for a favorable prognosis [16, 17]. Extensive reduction arterioplasty and translocation of the pulmonary artery anterior to the aorta are two common operative approaches to reduce the compression [18, 19]. In our cohort 74% (23/31) of the children were diagnosed in infancy (age 1–12 months), and the majority of these children (19/23) underwent surgery in infancy with pulmonary angioplasty.

The tracheobronchial compression seen with absent pulmonary valve, especially absent pulmonary valve syndrome, is different from that noted with vascular rings and left pulmonary artery sling [20–22]. The tracheobronchial compression seen with vascular rings usually relates to the aorta and aberrant branches, while the compression with left pulmonary artery sling is related to the aberrant course of the left pulmonary artery and is often associated with complete cartilaginous tracheal and bronchial rings. Although absent pulmonary valve syndrome is frequently associated with tracheobronchial compression, as seen in our case review, the morbidity and mortality are lower than that for left pulmonary artery sling [22].

The prognosis of absent pulmonary valve is dependent not only on the cardiac defects but largely on the associated tracheobronchial compression. If children with absent pulmonary valve have early surgery with correction of the cardiac defects and pulmonary angioplasty or translocation, the prognosis is good [17, 23].

## Conclusion

In children with absent pulmonary valve, marked dilatation of the pulmonary arteries can cause significant morbidity and mortality from adjacent tracheobronchial compression. Multi-slice CT in the axial plane and with coronal 3-D reconstruction can accurately depict areas of significant compression adjacent to markedly dilated pulmonary arteries and associated lung lesions, helping to direct the surgeon prior to pulmonary reduction angioplasty or translocation.
